# Association between serum 5-methyltetrahydrofolate and homocysteine in Chinese hypertensive participants with different MTHFR C677T polymorphisms: a cross-sectional study

**DOI:** 10.1186/s12937-022-00786-w

**Published:** 2022-05-13

**Authors:** Yu Cheng, Shuai Liu, Duo Chen, Yiman Yang, Qiongyue Liang, Ya Huo, Ziyi Zhou, Nan Zhang, Zhuo Wang, Lishun Liu, Yun Song, Xiangyi Liu, Yong Duan, Xiuwen Liang, Bingjie Hou, Binyan Wang, Genfu Tang, Xianhui Qin, Fangrong Yan

**Affiliations:** 1 State Key Laboratory of Natural Medicines, Research Center of Biostatistics and Computational Pharmacy, China Pharmaceutical University, Nanjing, 210000 China; 2 Beijing Advanced Innovation Center for Food Nutrition and Human Health, College of Food Science and Nutritional Engineering, China Agricultural University, Beijing, China; 3 Department of Cardiology, Peking University First Hospital, Beijing, China; 4 Institute of Biomedicine, Anhui Medical University, Hefei, China; 5 BeiJing TongRen Hospital, CMU, Beijing, China; 6 Yunnan Key Laboratory of Laboratory Medicine, Kunming, China; 7 Department of Clinical Laboratory, the First Affiliated Hospital of Kunming Medical University, Kunming, China; 8 China Mongolia Hospital of Hulunbuir, Inner Mongolia, China; 9 The Fourth Central Hospital of Baoding City, Hebei Baoding, China; 10 Shenzhen Evergreen Medical Institute, Shenzhen, China; 11 School of Health Administration, Anhui Medical University, Hefei, China; 12 Division of Nephrology, Nanfang Hospital, Southern Medical University, National Clinical Research Center for Kidney Disease; State Key Laboratory of Organ Failure Research; Guangdong Provincial Institute of Nephrology, Guangdong Provincial Key Laboratory of Renal Failure Research, Guangzhou Regenerative Medicine and Health Guangdong Laboratory, Guangzhou, 510000 China

**Keywords:** 5-methyltetrahydrofolate, Homocysteine, MTHFR C677T

## Abstract

**Background and aims:**

Clarifying the association between 5-methyltetrahydrofolate and homocysteine and the effect pattern of methylene tetrahydrofolate reductase (MTHFR C677T) may contribute to the management of homocysteine and may serve as a significant reference for a randomized controlled trial of 5-methyltetrahydrofolate intervention. This study aimed to reveal the association between these two biochemical indices.

**Methods:**

Study population was drawn from the baseline data of the China Stroke Primary Prevention Trial (CSPPT), including 2328 hypertensive participants. 5-methyltetrahydrofolate and homocysteine were determined by stable-isotope dilution liquid chromatography-tandem mass spectrometry and automatic clinical analyzers, respectively. MTHFR C677T polymorphisms were detected using TaqMan assay. Multiple linear regression was performed to evaluate the association between serum 5-methyltetrahydrofolate and homocysteine.

**Results:**

There was a significant inverse association between 5-methyltetrahydrofolate and homocysteine when 5-methyltetrahydrofolate was ≤ 10 ng/mL, and this association was modified by MTHFR C677T (per 1-ng/mL increment; All: *β* = − 0.50, *P* <  0.001; CC: *β* = − 0.14, *P* = 0.087; CT: *β* = − 0.20, *P* = 0.011; TT: *β* = − 1.19, *P* <  0.001). Moreover, the decline in trend in genotype TT participants was stronger than in genotype CC participants (*P* for difference <  0.001) and genotype CT participants (*P* for difference <  0.001), while there was no significant difference between genotype CC and genotype CT participants (*P* for difference = 0.757).

**Conclusions:**

Our data showed a non-linear association between serum homocysteine and 5-methyltetrahydrofolate among Chinese hypertensive adults, however, it could be inversely linearly fitted when serum 5-methyltetrahydrofolate was ≤ 10 ng/mL, and this association was modified by MTHFR C677T.

**Supplementary Information:**

The online version contains supplementary material available at 10.1186/s12937-022-00786-w.

## Background

Homocysteine (Hcy), a sulfur-containing amino acid, is an intermediate product in the normal metabolism from methionine to cysteine. It has been shown that hyperhomocysteinemia (blood concentration of Hcy > 15 *μ* mol/L) is significantly correlated with cardiovascular disease [1–3]. Evidence from the CSPPT (China Stroke Primary Prevention Trial) showed that appropriate supplementation of folic acid can significantly reduce Hcy concentrations, thereby reducing the risk of stroke and delaying the progression of chronic kidney disease (CKD) among patients with mild-to-moderate CKD [4].

Although numerous randomized controlled trials investigated the efficacy of lowering Hcy with folic acid for cardiovascular disease [4–6], a level above a certain threshold dose (e.g., after the first tablet of 200 μg folic acid; or 100 μg/day folic acid supplement for 10 days) results in accumulation of unmetabolized folate [7, 8], which could affect the normal homeostatic regulation of folate and has been reported to be associated with reduced natural killer cell cytotoxicity [9]. 5-methyltetrahydrofolate (5-MeTHF), the major bioactive form of folate metabolism, might be a potential alternative to folic acid because of its advantages: it does not require an activation process (folate itself is not active and has to undergo several metabolic steps within the cell in order to be converted into the metabolically active 5-methyltetrahydrofolate form [10]), avoids the influence of low enzymatic activity or genetic defects, and is free of the possible accumulation of unmetabolised folate.

In terms of chemical mechanism, methylenetetrahydrofolate reductase (MTHFR) is a crucial enzyme in folic acid metabolism [11]. A common mutation in the gene encoding the MTHFR enzyme is a C to T substitution at bp 677 (MTHFR C677T). The thermolabile mutated phenotype (TT) has a residual activity of about 50% of that of the wild-type controls (CC), resulting in elevated levels of Hcy [12].

Studies have demonstrated significantly negative associations between 5-MeTHF and Hcy among different populations [13–15]. However, these studies were not designed to investigate these associations directly, the results based on these studies are limited by intervention time (several hours to 1 year), sample size (44 to 228), and participant characteristics (pregnant women, young adult, colorectal cancer patient), thus, current results are not adequate to evaluate the association between 5-methyltetrahydrofolate and homocysteine. Moreover, the influence of MTHFR C677T on the relationship between 5-MeTHF and Hcy is overlooked in the majority of the current studies. It is estimated that 26% of the world’s population suffers from hypertension and this is expected to increase to 29% by 2025 [16]. Therefore, it is of high practical significance to evaluate the relationship between 5-MeTHF and Hcy in hypertensive populations.

Overall, clarifying the association between 5-MeTHF and Hcy and the effect pattern of MTHFR C677T may contribute to the management of Hcy, help to verify the possible single-dose supplementation threshold of 5-methyltetrahydrofolate, and guide the dose adjustment in different MTHFR C677T genotypes, which may serve as a significance reference for the precise supplementation and intervention of 5-MeTHF. We conducted a cross-sectional study (a sub-study of the CSPPT) in a Chinese rural population with hypertension to reveal the relationship between 5-MeTHF and Hcy and to evaluate the effect pattern of the MTHFR C677T polymorphism.

## Methods

### Data availability statement

The parent study (the China Stroke Primary Prevention Trial, CSPPT) was approved by the Ethics Committee of the Institute of Biomedicine, Anhui Medical University, Hefei, China (federal-wide assurance number: FWA00001263). All participants provided written, informed consent. The data, analytic methods and study materials that support the findings of this study will be available from the corresponding author on request after the request is submitted and formally reviewed and approved by the Ethics Committee of the Institute of Biomedicine, Anhui Medical University.

### Study population and design (parent design, CSPPT)

The methods and major results of the CSPPT have been reported elsewhere [17]. Briefly, the CSPPT was a multi-community, randomized, double-blinded, controlled trial conducted from 19 May 2008 to 24 August 2013 in 32 communities in China. Eligible participants were men and women aged 45–75 y with hypertension, defined as seated, resting systolic blood pressure (SBP) ≥ 140 mmHg or/and diastolic blood pressure (DBP) ≥ 90 mmHg at both the screening and recruitment visit, or who were taking any antihypertensive medication. The major exclusion criteria included a history of physician-diagnosed stroke, myocardial infarction, heart failure, postcoronary revascularization, or congenital heart disease. A total of 20,702 eligible participants, stratified by the methylenetetrahydrofolate reductase (MTHFR) C677T genotype (CC, CT or TT), were randomly assigned in a 1:1 ratio to 1 of 2 treatment groups: a daily oral dose of 1 tablet containing 10 mg enalapril and 0.8 mg folic acid (the enalapril + folic acid group) or a daily oral dose of 1 tablet containing 10 mg enalapril only (the enalapril group). Participants were engaged in follow-up every 3 months.

### Cross-sectional study (sub study)

Using data from the CSPPT, we established a sub-database of 2590 participants, including all the 1326 incident stroke, cancer, or all-cause mortality cases at the exit site visit matched with 1264 corresponding controls. Controls were randomly chosen from the baseline CSPPT participants who did not develop the corresponding end points during the follow-up, and were matched with the cases on a 1:1 ratio for age (± 1 year), sex, treatment group and study site. Our final analysis included 2328 participants, after excluding 238 participants with missing data of 5-MeTHF (*n* = 204) or Hcy (*n* = 34), and 24 participants with extreme value of 5-MeTHF (Supplemental Fig. 1).

### Laboratory assays

Serum 5-methyltetrahydrofolate concentration was measured by stable-isotope dilution liquid chromatography-tandem mass spectrometry using 4500MD (AD SCIEX) in a commercial lab (Shenzhen Tailored Medical Laboratory, China). Serum folate, vitamin B12 and vitamin D3 were measured by a commercial laboratory with the use of a chemiluminescent immunoassay (New Industrial). Serum homocysteine, fasting lipids, and glucose levels were measured using automatic clinical analyzers (Beckman Coulter) at the core laboratory of the National Clinical Research Center for Kidney Disease, Nanfang Hospital, Guangzhou, China. MTHFR C677T (rs1801133) polymorphisms were detected on an ABI Prism 7900HT sequence detection system (Life Technologies) using the TaqMan assay.

### Statistical analysis

Baseline characteristics were presented as median (interquartile range, IQR) for continuous variables and as *n* (%) for categorical variables. Differences in baseline characteristics between different genotypes were compared through the use of a Kruskal test for continuous variables and a chi-square test for categorical variables. *β* s of Hcy concentration in relation to serum 5-MeTHF concentration were calculated through the use of multiple linear regression models, with adjustment for sex, age, study site, body mass index (BMI), systolic blood pressure (SBP), diastolic blood pressure (DBP), estimated glomerular filtration rate (eGFR), total cholesterol (TC), triglycerides (TG), high-density lipoprotein cholesterol (HDL-C), vitamin B12, vitamin D3, fasting glucose, folate, smoking (never, ever, and current), alcohol drinking (never, ever, and current) and MTHFR C677T polymorphisms (CC, CT, and TT) at baseline.

A 2-tailed *P* <  0.05 was considered to be statistically significant in all analyses. R software (version 3.6.0) was used for all statistical analyses.

## Results

### Study participants and baseline characteristics

As shown in the flow chart (Supplemental Fig. 1), 2328 participants were included in the final analysis of this study. Baseline characteristics of study participants stratified by MTHFR C677T are shown in Table 1. Genotype CT participants and genotype TT participants had higher levels of Hcy concetrations, DBP, BMI, TC and fasting glucose but lower levels of 5-MeTHF concentrations, vitamin B12 and folate compared with genotype CC participants at baseline. Similar trends were found in males and females (Supplemental Table 1, Supplemental Table 2).

**Table 1:** Baseline characteristics of the study participants stratified by MTHFR C677T

Characteristics	Total (***N*** = 2328)	MTHFR C677T			***P*** Value
		CC (***N*** = 691)	CT (***N*** = 1129)	TT (***N*** = 580)	
**Age, years**	63.2 (57.5, 69.1)	64.3 (57.5, 70.1)	62.8 (57.5, 68.8)	63.4 (57.6, 68.3)	0.060
**Male,** ***n*** **(%)**	1127 (48.3)	290 (46.8)	561 (49.7)	275 (47.2)	0.437
**BMI, kg/m**^**2**^	24.2 (21.8, 26.9)	23.7 (21.2, 26.6)	24.3 (22.0, 27.0)	24.5 (21.9, 27.3)	0.002
**SBP, mmHg**	166.7 (156.0, 180.7)	166.0 (156.7, 180.0)	167.3 (155.3, 181.3)	167.3 (156.0, 180.8)	0.976
**DBP, mmHg**	93.3 (86.0, 100.7)	90.7 (83.3, 100.0)	94.0 (86.0, 100.7)	94.7 (87.3, 100.7)	< 0.001
**eGFR, mL/min/1.73m**^**2**^	92.2 (83.5, 99.1)	91.7 (81.7, 99.0)	92.7 (84.2, 99.0)	92.0 (83.9, 99.5)	0.286
**TC, mmol/L**	5.4 (4.7, 6.2)	5.3 (4.6, 6.1)	5.4 (4.7, 6.2)	5.5 (4.8, 6.3)	0.011
**TG, mmol/L**	1.4 (1.0, 1.9)	1.3 (1.0, 1.9)	1.4 (1.0, 1.9)	1.3 (1.0, 1.9)	0.754
**HDL-C, mmol/L**	1.3 (1.1, 1.6)	1.3 (1.1, 1.6)	1.3 (1.1, 1.6)	1.3 (1.1, 1.5)	0.987
**Vitamin B12, pg/mL**	382.9 (318.2, 478.5)	391.2 (323.6, 485.2)	388.0 (323.2, 485.2)	367.6 (307.7, 453.3)	0.001
**Vitamin D3, ng/mL**	19.1 (13.7, 25.3)	19.1 (14.3, 25.1)	19.2 (13.6, 25.1)	18.8 (13.5, 24.7)	0.145
**Glu, mmol/L**	5.4 (4.9, 6.1)	5.3 (4.7, 6.0)	5.4 (4.9, 6.2)	5.4 (5.0, 6.2)	0.001
**Folate, ng/mL**	7.8 (5.3, 10.4)	9.0 (6.2, 11.3)	8.0 (5.5, 10.4)	6.2 (4.6, 9.0)	< 0.001
**5-MeTHF, ng/mL**	5.4 (3.1, 9.0)	5.5 (3.1, 9.0)	5.6 (3.2, 9.5)	4.8 (2.8, 8.2)	0.002
**Hcy,** *μ* **mol/L**	13.4 (11.1, 16.8)	12.6 (10.6, 15.2)	13.0 (11.0, 15.5)	16.4 (12.8, 24.9)	< 0.001

Values are median (interquartile range) or *n* (%). Differences in baseline characteristics were compared using chi-square test for categorical variables and Kruskal test for continuous variables. *BMI* body-mass index, *SBP* systolic blood pressure, *DBP* diastolic blood pressure, *eGFR* estimated glomerular filtration rate, *TG* triglycerides, *TC* total cholesterol, *HDL-C* high-density lipoprotein cholesterol, *Glu* glucose, *5-MeTHF* 5-methyltetrahydrofolate, *Hcy* homocysteine

### Association of serum 5-MeTHF and Hcy and stratification by MTHFR C677T

**Fig. 1:**
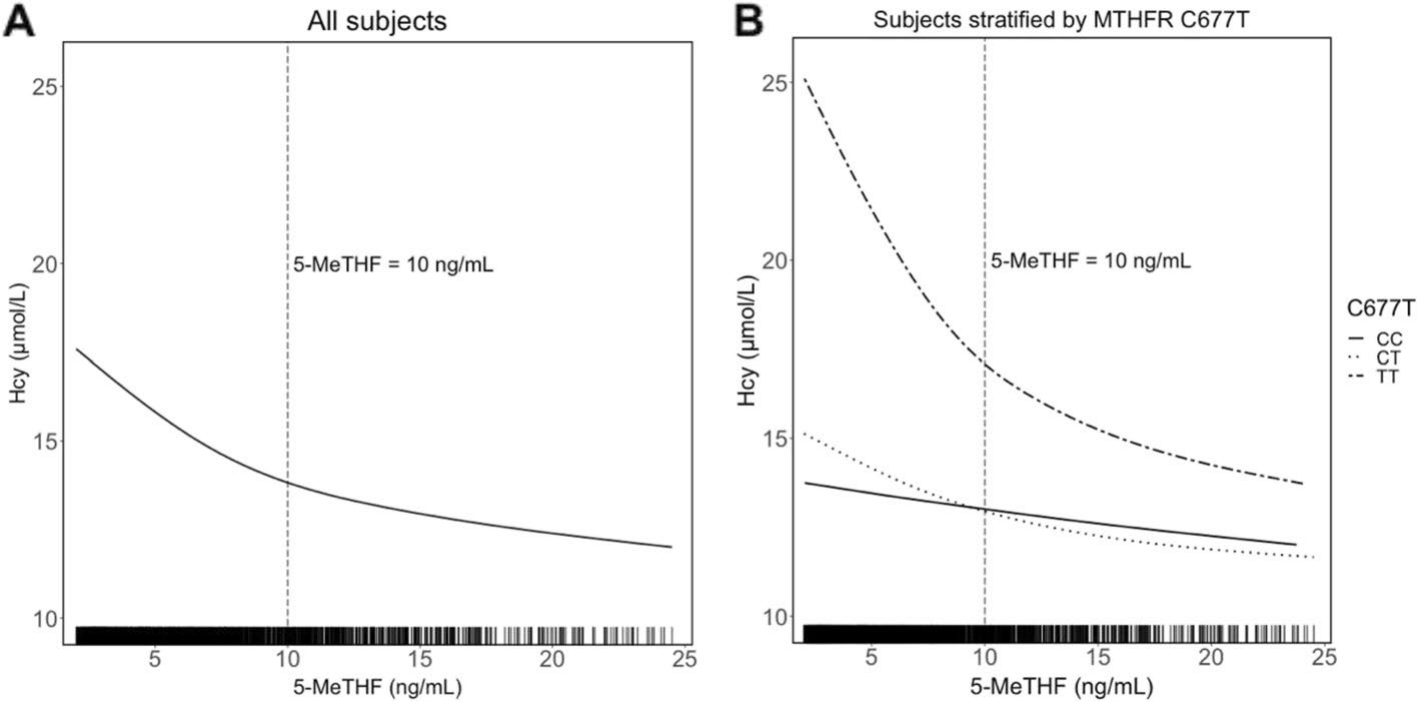
Association of Hcy levels and 5-MeTHF levels among all participants (**A**) and stratified by MTHFR C677T (**B**). Panel A was adjusted for sex, age, study site, MTHFR C677T, BMI, systolic blood pressure, diastolic blood pressure, estimated glomerular filtration rate, total cholesterol, triglycerides, high-density lipoprotein cholesterol, vitamin B12, vitamin D3, fasting glucose, folate, smoking and drinking at baseline. Panel B was adjusted for the variables in panel A but removed MTHFR C677T

Overall, there existed an apparent inverse association between serum 5-MeTHF and Hcy during the baseline period, whether among all participants (Fig. 1A) or stratified by MTHFR C677T (Fig. 1B; solid, dotted and two-dash lines represent for genotype CC, CT and TT, respectively), but the association plateaued when the 5-MeTHF concentration was over 10 ng/mL (Supplemental Table 3). Thus, the 2328 participants were divided into two subsets, one of which had concentrations of 5-MeTHF ≤ 10 ng/mL, while the other subset had concentrations over 10 ng/mL. Multiple linear regression was performed on both subsets (Supplemental Table 4). The results of the former subset were displayed in Table 2.

**Table 2:** Multiple linear regression model of Hcy on level of 5-MeTHF in all participants and stratified by MTHFR C677T genotype (5-MeTHF ≤ 10 ng/mL only)

5-MeTHF (ng/mL)	N	Hcy	Adjusted model	
		Median (IQR)	β (95% CI)	***P*** Value
**All Participants**				
Continuous	1840	13.8 (11.4, 17.5)	−0.50 (− 0.69, − 0.31)	< 0.001
Quartiles				
Q1 (< 2.7)	460	15.3 (12.7, 20.1)	*Ref*	
Q2 (2.7 - < 4.4)	460	14.6 (11.7, 18.1)	−2.18 (−3.32, −1.03)	< 0.001
Q3 (4.4 - < 6.4)	459	13.3 (11.3, 16.7)	−2.51 (− 3.71, − 1.31)	< 0.001
Q4 (≥ 6.4)	461	12.5 (10.5, 15.2)	−3.62 (−4.87, − 2.38)	< 0.001
*P* for trend				< 0.001
**Genotype CC**				
Continuous	485	12.9 (10.8, 15.4)	−0.14 (−0.30, 0.02)	0.087
Quartiles				
Q1 (< 2.8)	121	14.0 (11.4, 16.2)	*Ref*	
Q2 (2.8 - < 4.5)	121	12.9 (11.1, 15.5)	−0.22 (− 1.20, 0.76)	0.662
Q3 (4.5 - < 6.5)	121	12.3 (10.5, 14.7)	−0.47 (− 1.48, 0.53)	0.353
Q4 (≥ 6.5)	122	12.5 (10.3, 14.6)	−0.98 (− 2.04, 0.08)	0.070
*P* for trend				0.061
**Genotype CT**				
Continuous	869	13.3 (11.2, 16.5)	−0.20 (− 0.35, − 0.05)	0.011
Quartiles				
Q1 (< 2.7)	217	14.7 (12.5, 18.1)	*Ref*	
Q2 (2.7 - < 4.5)	217	14.1 (11.5, 16.9)	−0.64 (− 1.54, 0.27)	0.170
Q3 (4.5 - < 6.5)	217	12.9 (11.2, 15.3)	−0.79 (− 1.73, 0.16)	0.105
Q4 (≥ 6.5)	218	12.0 (10.1, 14.5)	−1.30 (−2.29, −0.30)	0.011
*P* for trend				0.013
**Genotype TT**				
Continuous	486	17.3 (13.3, 26.1)	−1.19 (− 1.77, −0.62)	< 0.001
Quartiles				
Q1 (< 2.7)	122	21.0 (15.8, 35.7)	*Ref*	
Q2 (2.7 - < 4.1)	121	17.9 (14.3, 27.4)	−5.92 (−9.46, −2.38)	0.001
Q3 (4.1 - < 6.2)	121	17.1 (13.2, 24.5)	−7.52 (−11.22, −3.81)	< 0.001
Q4 (≥ 6.2)	122	14.1 (11.7, 19.1)	−9.02 (−12.84, −5.20)	< 0.001
*P* for trend				< 0.001

Adjusted for sex, age, study site, BMI, systolic blood pressure, diastolic blood pressure, estimated glomerular filtration rate, total cholesterol, triglycerides, high-density lipoprotein cholesterol, vitamin B12, vitamin D3, fasting glucose, folate, smoking and drinking at baseline

A 1-ng/mL increment in 5-MeTHF concentration was associated with a 0.50- *μ* mol/L decrease in Hcy concentration (95% CI: − 0.69, − 0.31; *P* <  0.001) among all participants. Consistently, when 5-MeTHF concentration was assessed as quartiles, the adjusted *β* s for participants in quartiles 2, 3 and 4 were − 2.18 (95% CI: − 3.32, − 1.03; *P* <  0.001), − 2.51 (95% CI: − 3.71, − 1.31; *P* <  0.001) and − 3.62 (95% CI: − 4.87, − 2.38; *P* <  0.001), respectively, when compared with those in quartile 1 (*P* for trend < 0.001) (Table 2).

Similarly, a significant, negative association was also found in genotype CT and TT participants, however, it was slightly not significant in genotype CC participants. A 1-ng/mL increase in 5-MeTHF concentration was associated with a 0.14- *μ* mol/L decrease (95% CI: − 0.30, 0.02; *P* = 0.087), a 0.20- *μ* mol/L decrease (95% CI: − 0.35, − 0.05; *P* = 0.011) and a 1.19- *μ* mol/L decrease (95% CI: − 1.77, − 0.62; *P* <  0.001) in Hcy concentration among CC, CT and TT genotype participants, respectively (Table 2). Moreover, the results showed that among participants whose serum 5-MeTHF was ≤10 ng/mL, the decline in trend in genotype TT participants was stronger than that in genotype CC and genotype CT participants, and there was no significant difference in the decline in trend between genotype CC and genotype CT participants. However, there were no significant differences in the decline in trend between genotype CC, genotype CT and genotype TT participants, whose serum 5-MeTHF concentrations were over 10 ng/mL (Supplemental Table 5).

Decrements in Hcy among CC genotype participants in quartile 2 (*P* = 0.662), quartile 3 (*P* = 0.353) and quartile 4 (*P* = 0.070) were not significant compared with quartile 1 when 5-MeTHF was assessed as quartiles. While a declining trend existed, this trend was not significant (*P* for trend = 0.061). Genotype CT participants showed a significant decrement in Hcy in quartile 4 (*P* = 0.013), but in genotype TT participants, when compared with quartile 1, all quartiles (all of the *P* values were ≤ 0.001) and the decline in trend were of statistical significance (*P* for trend < 0.001) (Table 2).

Considering the potential heterogeneity between participants with and without outcomes (incident stroke, cancer or all-cause mortality), we performed a sensitivity analysis on these two sub-groups respectively. The overall association was consistent with the above results regardless of the subgroup (Supplemental Table 6, Supplemental Table 7).

There were no declining trend results found among the participants whose 5-MeTHF concentration was over 10 ng/mL, either in all participants or stratified by MTHFR C677T (Supplemental Table 4).

### Modification of MTHFR C677T on the association between 5-MeTHF and Hcy

Stratified analyses were performed to assess the association between serum 5-MeTHF (≤ 10 ng/mL, as a continuous variable) and Hcy (Supplemental Table 8) in various subgroups including MTHFR C677T. A significantly stronger negative association between serum 5-MeTHF and Hcy was observed in genotype TT participants (per increment, *β*: -1.19; 95% CI: − 1.77, − 0.62; *P* <  0.001) than in genotype CC participants (per increment, *β*: -0.14; 95% CI: − 0.30, 0.02; *P* = 0.087) and genotype CT participants (per increment, *β*: -0.20; 95% CI: − 0.35, − 0.05; *P* = 0.011). However, there was no significant difference between genotype CC and genotype CT participants (Supplemental Table 5). Further, in participants with lower vitamin B12 ≤ 381.4 pg/mL (per increment, *β*: -0.75; 95% CI: − 1.08, − 0.41; *P* <  0.001) a significantly stronger negative association was observed than in those with vitamin B12 > 381.4 pg/mL (per increment, *β*: -0.27; 95% CI: − 0.46, − 0.07; *P* = 0.007; *P* for interaction = 0.007).

None of the other variables, including age, SBP, eGFR, vitamin D3 and fasting glucose over the baseline period showed significant effect modification on the association between serum 5-MeTHF and Hcy (*P* for interaction > 0.05 for all of these stratified variables).

### Interaction effect analysis of 5-MeTHF and MTHFR C677T on Hcy concentration

Participants whose 5-MeTHF concentration was ≤ 10 ng/mL, were quartered according to their 5-MeTHF concentration (i.e., Q1, < 2.7 ng/mL; Q2, 2.7 – < 4.4 ng/mL; Q3, 4.4 – < 6.4 ng/mL; Q4, ≥ 6.4 ng/mL) and were stratified by MTHFR C677T. Moreover, genotype CC participants in quartile 1 were set as reference. Genotype TT participants in Q1 ~ Q4 (all of the adjusted *P* values were <  0.05) showed significant positive coefficients. In particular, when comparing Q4 in genotype TT participants with Q1 in genotype CC participants (reference), the coefficient was 3.46 (95% CI: 1.17, 5.75; *P* = 0.003). However, there were no significant differences in Q2 ~ Q4 among genotype CC participants and Q1 ~ Q4 among genotype CT participants when compared with Q1 among genotype CC particpants (all of the adjusted *P* values were > 0.05), and the *P* value for the trend test was < 0.001 (Table 3).

**Table 3:** Interaction effect between serum 5-MeTHF (≤ 10 ng/mL) concentration and MTHFR C677T on Hcy concentration

Subgroups (5-MeTHF ng/mL)	N	Crude Model		Adjusted Model	
		β (95% CI)	***P*** Value	β (95% CI)	***P*** Value
**Genotype CC**					
Q1 (< 2.7)	115	*Ref*		*Ref*	
Q2 (2.7 - < 4.4)	127	−0.74 (−3.10, 1.61)	0.536	0.35 (−1.79, 2.51)	0.745
Q3 (4.4 - < 6.4)	119	−1.1 7 (−3.57, 1.22)	0.337	−0.04 (−2.25, 2.17)	0.973
Q4 (≥ 6.4)	124	−1.12 (−3.49, 1.25)	0.355	−0.01 (−2.23, 2.22)	0.996
**Genotype CT**					
Q1 (< 2.7)	213	1.53 (−0.59, 3.65)	0.157	1.21 (−0.72, 3.15)	0.217
Q2 (2.7 - < 4.4)	204	0.55 (−1.59, 2.68)	0.615	0.54 (−1.41, 2.51)	0.583
Q3 (4.4 - < 6.4)	226	−0.37 (−2.47, 1.73)	0.729	0.52 (−1.44, 2.49)	0.602
Q4 (≥ 6.4)	226	−1.39 (−3.49, 0.71)	0.195	0.11 (−1.88, 2.11)	0.910
**Genotype TT**					
Q1 (< 2.7)	132	15.80 (13.67, 18.14)	< 0.001	14.77 (12.63, 16.92)	< 0.001
Q2 (2.7 - < 4.4)	129	8.16 (5.81, 10.51)	< 0.001	8.00 (5.83, 10.17)	< 0.001
Q3 (4.4 - < 6.4)	114	7.07 (4.65, 9.49)	< 0.001	7.22 (4.94, 9.50)	< 0.001
Q4 (≥ 6.4)	111	2.60 (0.16, 5.03)	0.037	3.46 (1.17, 5.75)	0.003
*P* for trend			< 0.001		< 0.001
*P* for interaction			< 0.001		< 0.001

Adjusted for sex, age, study site, BMI, systolic blood pressure, diastolic blood pressure, estimated glomerular filtration rate, total cholesterol, triglycerides, high-density lipoprotein cholesterol, vitamin B12, vitamin D3, fasting glucose, folate, smoking and drinking at baseline

## Discussion

In this cross-sectional study derived from the CSPPT, we demonstrated a significant inverse association between serum 5-MeTHF concentration and Hcy concentration among a Chinese hypertensive population, and this association was regulated by MTHFR C677T. Moreover, the inverse association was mainly found in participants whose serum 5-MeTHF was ≤10 ng/mL.

Former studies have demonstrated the equivalent or better bioavailability of 5-MeTHF in elevating blood concentration of 5-MeTHF and safety compared with folate [18–20], however, the association between 5-MeTHF and Hcy was slightly different in different studies. A recent study conducted in a Japanese pregnant cohort (*n* = 434) stated a significant, negative association between serum 5-MeTHF and Hcy at each blood sampling period (early pregnancy, late pregnancy, at birth, and cord blood) [13]. Agata et al. recruited 144 participants aged 19–64 years and found no association between plasma 5-MeTHF and Hcy but found a weak association between whole blood 5-MeTHF and Hcy (*r* = − 0.18; *P* = 0.043) [21]. However, Ji et al. found a significant, negative relationship between whole blood 5-MeTHF and Hcy in healthy participants aged from 20 to 25 years (*n* = 44; *r* = − 0.52; *P* <  0.001) [14], and Elizabeth et al. found a significant, negative relationship between rectal mucosal 5-MeTHF and plasma Hcy in bowel cancer individuals (*n* = 95; *r* = − 0.16; *P* = 0.017) [15]. Differences in the results between these studies might be attributed to the heterogeneity of the population and the sample size. Moreover, vitamin B12 and vitamin D3, which have been reported as a modifier of methionine synthase reductase (MTR) and a reduced folate carrier, respectively [22, 23], were not taken into account in the analysis either. Our current study focused on a Chinese middle-aged and elderly hypertensive population and the inverse association derived from this study was consistent with former studies, which may indicate that an inverse association generally exists among this population.

Compared with former studies, our study provides two novel insights. First, this was the largest study designed to evaluate the association between serum 5-MeTHF and Hcy, and it identified the turning point of serum 5-MeTHF to be at 10 ng/mL, where a significant inverse association between 5-MeTHF and Hcy was shown when serum 5-MeTHF concetration was ≤10 ng/mL. From the perspective of one-carbon metabolism, 5-MeTHF provides a methyl group and homocysteine is remethylated to methionine in the presence of methionine synthase and further converted to S-adenosine methionine (SAM) [22]. However, when serum 5-MeTHF concentration comes to a certain level, methionine synthase approaches its maximum enzyme activity accumulation, which may account for the inverse association between serum 5-MeTHF and Hcy merely when serum 5-MeTHF was ≤10 ng/mL. Second, to our knowledge, this was the first study to qualify the determination of MTHFR C677T on the relationship between serum 5-MeTHF and Hcy, and revealed an effect pattern. The initial report by Frosst et al. demonstrated significantly elevated plasma Hcy levels in the homozygous MTHFR C677T mutant [12]. This association was further proven to be presented only in individuals with low folate status [24]. The results derived from our study were similar with these conclusions. When the participants whose serum 5-MeTHF was ≤10 ng/mL were stratified by MTHFR C677T, it was apparent that serum Hcy concentration in genotype CC participants was not sensitive to the serum 5-MeTHF. By contrast, genotype CT and TT participants showed a significant decline in trend. The possible mechanisms by which genotype TT participants declined more dramatically than genotype CT and CC participants may be due to the decrease in MTHFR enzymatic activity, leading to the accumulation of Hcy [25], while higher levels of Hcy creates more sensitivity to changes in serum 5-MeTHF. Further studies are needed to verify this hypothesis. Previous studies have demonstrated that folate and vitamin B12 status can affect the enzyme activity of MTHFR and methionine synthase, respectively [22, 26]. In our current study, genotype TT participants had relatively low levels of folate, low vitamin B12 concentrations and higher diastolic blood pressure compared with genotype CC and genotype CT participants, which may partly mask the association between serum 5-MeTHF and Hcy [27, 28]. We observed that vitamin B12 status modified but did not alter this association. We conjecture that although our current study findings suggest that these factors did not distort the relation, they may have partly attenuated or enhanced the decline in trend. Accordingly, our study indicated a dose-response relation of 5-MeTHF with Hcy, and pointed out potential factors that might affect the multidimensional process of 5-MeTHF supplementation.

A few limitations of our study should be addressed. First, our study was a baseline cross-sectional and a secondary analysis of the CSPPT. This study was not included in the original protocol of the trial, nor in the clinical trial registration. Second, the current study did not take the polymorphism of methionine synthase into account. Third, our current study was conducted in hypertensive patients, thus the generalizability of the results to adults without hypertension remains to be examined. Overall, our findings were just hypothesis-generating. All reported results should be further investigated and confirmed in future studies. If further confirmed, our findings may lend further support that maintaining a relatively higher serum 5-MeTHF status may be helpful for the prevention of hyperhomocysteinemia or relative cardiovascular diseases triggered by hyperhomocysteinemia in hypertensive patients regardless of polymorphisms of MTHFR C677T.

## Conclusions

In summary, a non-linear association was found between serum 5-MeTHF and Hcy in Chinese hypertensive participants, however, serum 5-MeTHF was significantly, inversely, and linearly associated with Hcy when serum 5-MeTHF was ≤10 ng/mL. Moreover, homozygous MTHFR C677T mutant participants significantly differed from those who were wild and heterozygous type.

## Supplementary Information

**Additional file 1.**

**Additional file 2.**

## Data Availability

The datasets used and analyzed during the current study could be available from the corresponding author on reasonable request.

## Abbreviations

5-MeTHF: 5-methyltetrahydrofolate
Hcy: Homocysteine
MTHFR: Methylene tetrahydrofolate reductase
CSPPT: China Stroke Primary Prevention Trial
CKD: Chronic kidney disease
IQR: Interquartile range
MTR: Methionine synthase reductase
SAM: S-adenosine methionine
