# Supplementary material for: Association between serum 5-methyltetrahydrofolate and homocysteine in Chinese hypertensive participants with different MTHFR C677T polymorphisms: a cross-sectional study
Source: Nutr J. 2022 May 13;21:29. doi: 10.1186/s12937-022-00786-w (PMC9102656; doi:10.1186/s12937-022-00786-w)
Supplement: Supplementary file 1 — Additional file 1. [file 12937_2022_786_MOESM1_ESM.docx]

CSPPT cohort

N = 20,702

Cross-sectional study

N = 2,590

2,352 participants

2,328 participants

**Supplemental Figure 1. Flow chart of the study participants**
