# Supplementary material for: Association between serum 5-methyltetrahydrofolate and homocysteine in Chinese hypertensive participants with different MTHFR C677T polymorphisms: a cross-sectional study
Source: Nutr J. 2022 May 13;21:29. doi: 10.1186/s12937-022-00786-w (PMC9102656; doi:10.1186/s12937-022-00786-w)
Supplement: Supplementary file 2 — Additional file 2. [file 12937_2022_786_MOESM2_ESM.docx]

**Supplemental Table 1. Baseline characteristics of the study participants stratified by MTHFR C677T (Males only)**

| **Characteristics** | **Total (N = 1203)** | **MTHFR C677T** | | | ***P* Value** |
| --- | --- | --- | --- | --- | --- |
|  |  | **CC (N = 329)** | **CT (N = 568)** | **TT (N = 306)** |  |
| **Age, years** | 64.3 (58.5, 69.7) | 66.4 (59.6, 71.0) | 63.2 (57.9, 69.4) | 64.2 (59.2, 68.8) | 0.004 |
| **BMI, kg/m^2^** | 23.6 (21.3, 26.0) | 23.0 (20.7, 25.7) | 23.9 (21.7, 26.0) | 23.7 (21.2, 26.3) | 0.008 |
| **SBP, mmHg** | 165.3 (154.7, 180.0) | 164.7 (155.3, 180.0) | 165.3 (154.0, 180.0) | 166.7 (152.7, 180.7) | 0.958 |
| **DBP, mmHg** | 94.0 (86.7, 101.3) | 91.3 (82.7, 100.7) | 95.3 (87.8, 102.0) | 95.7 (88.7, 100.7) | 0.018 |
| **eGFR, mL/min/1.73m^2^** | 90.8 (82.1, 97.4) | 90.0 (80.4, 96.9) | 91.1 (83.0, 98.0) | 90.6 (82.3, 97.4) | 0.432 |
| **TG, mmol/L** | 1.2 (1.0, 1.6) | 1.2 (0.9, 1.6) | 1.2 (1.0, 1.7) | 1.2 (0.9, 1.6) | 0.692 |
| **TC, mmol/L** | 5.3 (4.6, 6.1) | 5.1 (4.5, 5.9) | 5.3 (4.6, 6.1) | 5.3 (4.7, 6.2) | 0.044 |
| **HDL-C, mmol/L** | 1.3 (1.1, 1.6) | 1.3 (1.1, 1.6) | 1.3 (1.1, 1.6) | 1.3 (1.1, 1.6) | 0.550 |
| **Vitamin B12, pg/mL** | 381.3 (314.7, 471.3) | 387.5 (320.2, 485.9) | 386.6 (318.8, 477.0) | 365.8 (304.9, 444.0) | 0.016 |
| **Vitamin D3, ng/mL** | 21.5 (15.3, 27.4) | 22.1 (15.2, 28.2) | 21.4 (15.1, 27.1) | 20.9 (15.5, 26.3) | 0.402 |
| **Glu, mmol/L** | 5.3 (4.8, 6.0) | 5.3 (4.8, 5.9) | 5.4 (4.9, 6.1) | 5.4 (4.9, 6.2) | 0.062 |
| **Folate, ng/mL** | 7.0 (4.9, 10.0) | 8.1 (5.8, 10.9) | 7.1 (5.2, 9.8) | 5.4 (4.2, 8.5) | < 0.001 |
| **5-MeTHF, ng/mL** | 4.8 (2.8, 7.9) | 5.0 (3.0, 8.1) | 4.7 (2.8, 8.1) | 4.3 (2.7, 7.0) | 0.030 |
| **Hcy,** $\mu$**mol/L** | 14.8 (12.4, 18.9) | 13.9 (11.8, 16.1) | 14.2 (12.2, 17.2) | 19.9 (14.8, 33.1) | < 0.001 |

Values are median (interquartile range). Differences in baseline characteristics were compared with the use of Kruskal test for continuous variables. BMI, body-mass index; SBP, systolic blood pressure; DBP, diastolic blood pressure; eGFR, estimated glomerular filtration rate; TG, triglycerides; TC, total cholesterol; HDL-C, high-density lipoprotein cholesterol; Glu, glucose; 5-MeTHF, 5-methyltetrahydrofolate; Hcy, homocysteine

**Supplemental Table 2. Baseline characteristics of the study participants stratified by MTHFR C677T (Females only)**

| **Characteristics** | **Total (N = 1125)** | **MTHFR C677T** | | | ***P* Value** |
| --- | --- | --- | --- | --- | --- |
|  |  | **CC (N = 290)** | **CT (N = 561)** | **TT (N = 274)** |  |
| **Age, years** | 62.3 (56.7, 68.2) | 62.4 (56.7, 68.1) | 62.4 (57.1, 68.4) | 62.0 (56.0, 68.1) | 0.757 |
| **BMI, kg/m^2^** | 25.1 (22.5, 27.6) | 24.9 (22.2, 27.2) | 25.1 (22.5, 27.7) | 25.5 (23.0, 27.9) | 0.066 |
| **SBP, mmHg** | 168.7 (156.7, 181.3) | 168.0 (157.3, 180.7) | 169.3 (156.7, 183.3) | 167.3 (157.7, 181.3) | 0.906 |
| **DBP, mmHg** | 91.3 (85.3, 100.0) | 90.0 (84.3, 99.3) | 91.3 (85.3, 100.0) | 94.3 (86.7, 100.0) | 0.013 |
| **eGFR, mL/min/1.73m^2^** | 93.8 (84.8, 100.5) | 93.1 (83.1, 100.9) | 94.2 (85.4, 100.3) | 93.7 (85.5, 100.9) | 0.621 |
| **TG, mmol/L** | 1.6 (1.2, 2.1) | 1.6 (1.2, 2.1) | 1.5 (1.2, 2.1) | 1.6 (1.2, 2.1) | 0.924 |
| **TC, mmol/L** | 5.6 (4.9, 6.4) | 5.5 (4.8, 6.3) | 5.6 (4.8, 6.4) | 5.7 (4.9, 6.5) | 0.225 |
| **HDL-C, mmol/L** | 1.3 (1.1, 1.5) | 1.3 (1.1, 1.5) | 1.3 (1.1, 1.5) | 1.3 (1.1, 1.5) | 0.538 |
| **B12, pg/mL** | 386.5 (323.6, 486.1) | 398.3 (326.0, 503.9) | 391.5 (325.8, 487.9) | 370.2 (313.0, 458.8) | 0.040 |
| **D3,** $\mu$**g/mL** | 17.3 (12.5, 22.5) | 17.4 (13.3, 23.5) | 17.4 (12.3, 22.4) | 16.8 (12.3, 21.8) | 0.277 |
| **Glu, mmol/L** | 5.4 (5.0, 6.2) | 5.3 (4.8, 6.2) | 5.5 (5.0, 6.3) | 5.5 (5.0, 6.2) | 0.020 |
| **Folate, ng/mL** | 8.6 (5.9, 10.8) | 9.3 (7.3, 11.6) | 8.6 (6.1, 11.0) | 7.0 (5.2, 9.4) | < 0.001 |
| **5-MeTHF, ng/mL** | 6.2 (3.5, 10.0) | 6.1(3.5, 10.3) | 6.5 (3.8, 10.8) | 5.7 (3.2, 9.7) | 0.037 |
| **Hcy,** $\mu$**mol/L** | 12.1 (10.2, 14.6) | 11.6 (9.8, 13.6) | 11.7 (10.0, 14.1) | 13.9 (11.2, 18.2) | < 0.001 |

Values are median (interquartile range). Differences in baseline characteristics were compared with the use of Kruskal test for continuous variables. BMI, body-mass index; SBP, systolic blood pressure; DBP, diastolic blood pressure; eGFR, estimated glomerular filtration rate; TG, triglycerides; TC, total cholesterol; HDL-C, high-density lipoprotein cholesterol; Glu, glucose; 5-MeTHF, 5-methyltetrahydrofolate; Hcy, homocysteine

**Supplemental Table 3. Cut-off test for all patients and different MTHFR C677T genotypes**

| **Contrast (High level compared with low level 5-MeTHF)** | ***P* for difference** |
| --- | --- |
| All participants | < 0.001 |
| Genotype CC | 0.139 |
| Genotype CT | 0.005 |
| Genotype TT | < 0.001 |

**Supplemental Table 4.** **Multiple linear regression model of Hcy on the level of 5-MeTHF by low and high 5-MeTHF level in all participants and stratified by MTHFR C677T genotype**

| **5-MeTHF**  **(ng/mL)** | **5-MeTHF ≤ 10 ng/mL** | | | | **5-MeTHF**  **(ng/mL)** | **5-MeTHF > 10 ng/mL** | | | |
| --- | --- | --- | --- | --- | --- | --- | --- | --- | --- |
|  | **N** | **Hcy** | **Adjusted model** | |  | **N** | **Hcy** | **Adjusted model** | |
|  |  | **Median (IQR)** | **β (95% CI)** | ***P* Value** |  |  | **Median (IQR)** | **β (95% CI)** | ***P* Value** |
| **All Participants PdewPaParticParticipants** |  |  |  |  | **All Subjects** |  |  |  |  |
| Continuous | 1840 | 13.8 (11.4, 17.5) | -0.50 (-0.69, -0.31) | < 0.001 | Continuous | 488 | 12.3 (10.3, 14.3) | 0.04 (-0.03, 0.12) | 0.258 |
| Quartiles |  |  |  |  | Quartiles |  |  |  |  |
| Q1 (< 2.7) | 460 | 15.3 (12.7, 20.1) | *Ref* |  | Q1 (< 11.5) | 122 | 12.8 (10.3, 14.6) | *Ref* |  |
| Q2 (2.7 - < 4.4) | 460 | 14.6 (11.7, 18.1) | -2.18 (-3.32, -1.03) | < 0.001 | Q2 (11.5 - < 13.8) | 122 | 12.2 (10.1, 14.7) | -0.26 (-1.49, 0.96) | 0.672 |
| Q3 (4.4 - < 6.4) | 459 | 13.3 (11.3, 16.7) | -2.51 (-3.71, -1.31) | < 0.001 | Q3 (13.8 - < 17.5) | 122 | 12.1 (10.3, 13.8) | 0.34 (-0.88, 1.57) | 0.581 |
| Q4 (≥ 6.4) | 461 | 12.5 (10.5, 15.2) | -3.62 (-4.87, -2.38) | < 0.001 | Q4 (≥ 17.5) | 122 | 12.1 (10.5, 13.9) | 0.27 (-1.00, 1.55) | 0.674 |
| *P* for trend |  |  |  | < 0.001 | *P* for trend |  |  |  | 0.478 |
| **Genotype CC** |  |  |  |  | **Genotype CC** |  |  |  |  |
| Continuous | 485 | 12.9 (10.8, 15.4) | -0.14 (-0.30, 0.02) | 0.087 | Continuous | 134 | 11.9 (10.3, 13.9) | 0.02 (-0.05, 0.09) | 0.633 |
| Quartiles |  |  |  |  | Quartiles |  |  |  |  |
| Q1 (< 2.8) | 121 | 14.0 (11.4, 16.2) | *Ref* |  | Q1 (< 11.7) | 34 | 11.8 (10.3, 14.8) | *Ref* |  |
| Q2 (2.8 - < 4.5) | 121 | 12.9 (11.1, 15.5) | -0.22 (-1.20, 0.76) | 0.662 | Q2 (11.7 - < 14.7) | 33 | 12.3 (10.5, 14.3) | 0.72 (-0.44, 1.88) | 0.225 |
| Q3 (4.5 - < 6.5) | 121 | 12.3 (10.5, 14.7) | -0.47 (-1.48, 0.53) | 0.353 | Q3 (14.7 - < 20.0) | 33 | 11.8 (10.0, 13.8) | 0.70 (-0.51, 1.90) | 0.259 |
| Q4 (≥ 6.5) | 122 | 12.5 (10.3, 14.6) | -0.98 (-2.04, 0.08) | 0.070 | Q4 (≥ 20.0) | 34 | 11.6 (10.5, 13.1) | 0.85 (-0.41, 2.11) | 0.190 |
| *P* for trend |  |  |  | 0.061 | *P* for trend |  |  |  | 0.225 |
| **Genotype CT** |  |  |  |  | **Genotype CT** |  |  |  |  |
| Continuous | 869 | 13.3 (11.2, 16.5) | -0.20 (-0.35, -0.05) | 0.011 | Continuous | 260 | 12.1 (10.2, 13.8) | 0.06 (-0.03, 0.16) | 0.183 |
| Quartiles |  |  |  |  | Quartiles |  |  |  |  |
| Q1 (< 2.7) | 217 | 14.7 (12.5, 18.1) | *Ref* |  | Q1 (< 11.6) | 65 | 12.8 (10.2, 13.8) | *Ref* |  |
| Q2 (2.7 - < 4.5) | 217 | 14.1 (11.5, 16.9) | -0.64 (-1.54, 0.27) | 0.170 | Q2 (11.6 - < 13.7) | 65 | 11.4 (9.5, 14.7) | 0.41 (-1.05, 1.86) | 0.585 |
| Q3 (4.5 - < 6.5) | 217 | 12.9 (11.2, 15.3) | -0.79 (-1.73, 0.16) | 0.105 | Q3 (13.7 - < 16.3) | 65 | 11.9 (10.4, 13.3) | -0.40 (-1.84, 1.03) | 0.581 |
| Q4 (≥ 6.5) | 218 | 12.0 (10.1, 14.5) | -1.30 (-2.29, -0.30) | 0.011 | Q4 (≥ 16.3) | 65 | 12.2 (10.4, 13.9) | 0.39 (-1.09, 1.88) | 0.605 |
| *P* for trend |  |  |  | 0.013 | *P* for trend |  |  |  | 0.899 |
| **Genotype TT** |  |  |  |  | **Genotype TT** |  |  |  |  |
| Continuous | 486 | 17.3 (13.3, 26.1) | -1.19 (-1.77, -0.62) | < 0.001 | Continuous | 94 | 13.4 (11.0, 16.4) | 0.10 (-0.21, 0.41) | 0.541 |
| Quartiles |  |  |  |  | Quartiles |  |  |  |  |
| Q1 (< 2.7) | 122 | 21.0 (15.8, 35.7) | *Ref* |  | Q1 (< 11.3) | 24 | 13.5 (10.8, 16.8) | *Ref* |  |
| Q2 (2.7 - < 4.1) | 121 | 17.9 (14.3, 27.4) | -5.92 (-9.46, -2.38) | 0.001 | Q2 (11.3 - < 13.7) | 23 | 14.1 (11.7, 18.8) | -1.15 (-5.83, 3.52) | 0.630 |
| Q3 (4.1 - < 6.2) | 121 | 17.1 (13.2, 24.5) | -7.52 (-11.22, -3.81) | < 0.001 | Q3 (13.7 - < 17.1) | 23 | 12.4 (11.0, 14.1) | 2.43 (-2.25, 7.12) | 0.313 |
| Q4 (≥ 6.2) | 122 | 14.1 (11.7, 19.1) | -9.02 (-12.84, -5.20) | < 0.001 | Q4 (≥ 17.1) | 24 | 13.3 (10.9, 15.3) | 2.07 (-2.42, 6.57) | 0.369 |
| *P* for trend |  |  |  | < 0.001 | *P* for trend |  |  |  | 0.215 |

Adjusted for sex, age, study site, BMI, systolic blood pressure, diastolic blood pressure, estimated glomerular filtration rate, total cholesterol, triglycerides, high-density lipoprotein cholesterol, vitamin B12, vitamin D3, fasting glucose, folate, smoking and drinking at baseline.

**Supplemental Table 5. Decline in trend test for different MTHFR C677T genotypes**

| **Contrast between different groups** | **5-MeTHF ≤ 10 ng/mL** | **5-MeTHF > 10 ng/mL** |
| --- | --- | --- |
|  | ***P* for difference** | ***P* for difference** |
| CC - CT | 0.757 | 0.952 |
| CC - TT | < 0.001 | 0.952 |
| CT - TT | < 0.001 | 0.845 |

**Supplemental Table 6. Sensitivity analysis for the association (Patients without outcomes, 5-MeTHF ≤ 10 ng/mL only)**

| **5-MeTHF (ng/mL)** | **N** | **Hcy** | **Adjusted model** | |
| --- | --- | --- | --- | --- |
|  |  | **Median (IQR)** | **β (95% CI)** | ***P* Value** |
| **All Participants** |  |  |  |  |
| Continuous | 907 | 13.6 (11.4, 17.6) | -0.45 (-0.73, -0.18) | 0.001 |
| Quartiles |  |  |  |  |
| Q1 (< 2.7) | 227 | 14.8 (12.6, 19.1) | *Ref* |  |
| Q2 (2.7 – < 4.4) | 226 | 13.9 (11.5, 17.8) | -1.71 (-3.37, -0.05) | 0.044 |
| Q3 (4.4 - < 6.4) | 227 | 13.4 (11.4, 17.0) | -2.13 (-3.85, -0.41) | 0.016 |
| Q4 (≥ 6.4) | 227 | 12.5 (10.2, 15.6) | -3.30 (-5.12, -1.49) | < 0.001 |
| *P* for trend |  |  |  | < 0.001 |
| **Genotype CC** |  |  |  |  |
| Continuous | 233 | 13.1 (10.9, 15.3) | -0.09 (-0.31, 0.14) | 0.453 |
| Quartiles |  |  |  |  |
| Q1 (< 2.8) | 58 | 13.9 (12.3, 15.2) | *Ref* |  |
| Q2 (2.8 - < 4.5) | 58 | 12.8 (10.9, 16.1) | 0.26 (-1.09, 1.62) | 0.704 |
| Q3 (4.5 - < 6.5) | 58 | 12.9 (10.2, 14.6) | 0.24 (-1.17, 1.66) | 0.737 |
| Q4 (≥ 6.5) | 58 | 12.6 (10.4, 14.6) | -0.62 (-2.12, 0.88) | 0.420 |
| *P* for trend |  |  |  | 0.450 |
| **Genotype CT** |  |  |  |  |
| Continuous | 445 | 12.9 (11.0, 15.7) | -0.62 (-0.27, 0.12) | 0.438 |
| Quartiles |  |  |  |  |
| Q1 (< 2.7) | 111 | 13.1 (12.3, 17.2) | *Ref* |  |
| Q2 (2.7 - < 4.5) | 111 | 13.1 (11.4, 16.5) | -0.62 (-1.74, 0.51) | 0.283 |
| Q3 (4.5 - < 6.5) | 111 | 12.6 (11.1, 14.9) | -0.65 (-1.81, 0.52) | 0.277 |
| Q4 (≥ 6.5) | 112 | 11.6 (9.9, 14.5) | -0.78 (-2.01, 0.46) | 0.217 |
| *P* for trend |  |  |  | 0.248 |
| **Genotype TT** |  |  |  |  |
| Continuous | 229 | 18.0 (13.6, 26.2) | -1.18 (-2.03, -0.32) | 0.008 |
| Quartiles |  |  |  |  |
| Q1 (< 2.7) | 57 | 21.8 (16.4, 32.1) | *Ref* |  |
| Q2 (2.7 - < 4.2) | 57 | 19.1 (14.7, 27.1) | -3.79 (-9.19, 1.62) | 0.171 |
| Q3 (4.2 - < 6.3) | 57 | 18.4 (14.0, 29.0) | -6.37 (-12.01, -0.73) | 0.028 |
| Q4 (≥ 6.3) | 58 | 14.9 (11.9, 19.8) | -8.60 (-14.38, -2.81) | 0.004 |
| *P* for trend |  |  |  | 0.003 |

**Supplemental Table 7. Sensitivity analysis for the association (Patients with outcomes, 5-MeTHF ≤ 10 ng/mL only)**

| **5-MeTHF (ng/mL)** | **N** | **Hcy** | **Adjusted model** | |
| --- | --- | --- | --- | --- |
|  |  | **Median (IQR)** | **β (95% CI)** | ***P* Value** |
| **All Participants** |  |  |  |  |
| Continuous | 933 | 14.1 (11.5, 17.5) | -0.54 (-0.81, -0.27) | < 0.001 |
| Quartiles |  |  |  |  |
| Q1 (< 2.7) | 233 | 15.7 (12.7, 21.3) | *Ref* |  |
| Q2 (2.9 - < 4.7) | 233 | 14.8 (11.8, 18.7) | -2.58 (-4.18, -0.98) | 0.002 |
| Q3 (4.7 - < 7.2) | 233 | 13.2 (11.3, 16.5) | -2.56 (-4.24, -0.88) | 0.003 |
| Q4 (≥ 7.2) | 234 | 12.7 (10.8, 14.9) | -3.89 (-5.62, -2.15) | < 0.001 |
| *P* for trend |  |  |  | < 0.001 |
| **Genotype CC** |  |  |  |  |
| Continuous | 275 | 12.6 (10.7, 15.4) | -0.17 (-0.40, 0.06) | 0.154 |
| Quartiles |  |  |  |  |
| Q1 (< 2.8) | 69 | 13.6 (10.4, 16.4) | *Ref* |  |
| Q2 (2.8 - < 4.5) | 68 | 12.9 (11.2, 15.3) | -0.24 (-1.60, 1.12) | 0.726 |
| Q3 (4.5 - < 6.5) | 69 | 12.1 (10.4, 14.5) | -0.44 (-1.87, 1.00) | 0.549 |
| Q4 (≥ 6.5) | 69 | 12.5 (10.1, 15.0) | -0.97 (-2.44, 0.50) | 0.193 |
| *P* for trend |  |  |  | 0.190 |
| **Genotype CT** |  |  |  |  |
| Continuous | 482 | 13.7 (11.6, 16.7) | -0.32 (-0.57, -0.07) | 0.012 |
| Quartiles |  |  |  |  |
| Q1 (< 2.7) | 121 | 15.4 (12.7, 19.3) | *Ref* |  |
| Q2 (2.7 - < 4.5) | 120 | 14.5 (11.8, 17.3) | -0.97 (-2.46, 0.51) | 0.197 |
| Q3 (4.5 - < 6.5) | 120 | 13.0 (11.2, 15.4) | -0.93 (-2.49, 0.64) | 0.243 |
| Q4 (≥ 6.5) | 121 | 12.5 (10.8, 14.7) | -1.92 (-3.53, -0.31) | 0.020 |
| *P* for trend |  |  |  | 0.029 |
| **Genotype TT** |  |  |  |  |
| Continuous | 273 | 16.8 (13.1, 26.0) | -1.13 (-1.93, -0.33) | 0.006 |
| Quartiles |  |  |  |  |
| Q1 (< 2.7) | 68 | 21.0 (13.1, 26.0) | *Ref* |  |
| Q2 (2.7 - < 4.2) | 68 | 17.5 (15.3, 37.6) | -6.61 (-11.48, -1.73) | 0.008 |
| Q3 (4.2 - < 6.3) | 68 | 16.7 (12.7, 24.1) | -7.30 (-12.37, -2.24) | 0.005 |
| Q4 (≥ 6.3) | 69 | 13.5 (11.2, 17.3) | -8.53 (-13.89, -3.18) | 0.002 |
| *P* for trend |  |  |  | 0.003 |

**Supplemental Table 8. The association between serum 5-MeTHF (≤ 10 ng/mL)and Hcy in various subgroups**

| **Subgroups** | **N** | **Adjusted model** | | |
| --- | --- | --- | --- | --- |
|  |  | **β (95% CI)** | ***P* value** | ***P* for interaction** |
| **Age, years** |  |  |  | 0.431 |
| $\leq$65 | 998 | -0.52 (-0.78, -0.26) | < 0.001 |  |
| > 65 | 842 | -0.48 (-0.76, -0.20) | < 0.001 |  |
| **BMI, kg/m^2^** |  |  |  | 0.028 |
| $\leq$24 | 921 | -0.33 (-0.61, -0.06) | 0.018 |  |
| > 24 | 919 | -0.69 (-0.96, -0.43) | < 0.001 |  |
| **SBP, mmHg** |  |  |  | 0.068 |
| $\leq$160 | 692 | -0.38 (-0.67, -0.10) | 0.008 |  |
| > 160 | 1148 | -0.62 (-0.83, -0.32) | < 0.001 |  |
| **DBP, mmHg** |  |  |  | < 0.001 |
| $\leq$90 | 798 | -0.23 (-0.45, -0.03) | 0.027 |  |
| > 90 | 1042 | -0.74 (-1.03, -0.44) | < 0.001 |  |
| **eGFR, mL/min/1.73m^2^** |  |  |  | 0.642 |
| $\leq$90 | 805 | -0.51 (-0.82, -0.20) | 0.001 |  |
| > 90 | 1035 | -0.50 (-0.74, -0.26) | < 0.001 |  |
| **Vitamin B12, pg/mL** |  |  |  | 0.007 |
| $\leq$ 381.4 | 920 | -0.75 (-1.08, -0.41) | < 0.001 |  |
| > 381.4 | 920 | -0.27 (-0.46, -0.07) | 0.007 |  |
| **Vitamin D3, ng/mL** |  |  |  | 0.524 |
| $\leq$19.7 | 929 | -0.46 (-0.74, -0.19) | < 0.001 |  |
| > 19.7 | 911 | -0.49 (-0.76, -0.22) | < 0.001 |  |
| **Glu, mmol/L** |  |  |  | 0.201 |
| $\leq$5.3 | 923 | -0.45 (-0.75, -0.16) | 0.003 |  |
| > 5.3 | 917 | -0.53 (-0.77, -0.28) | < 0.001 |  |
| **MTHFR C677T** |  |  |  | < 0.001 |
| CC | 485 | -0.14 (-0.30, 0.02) | 0.087 |  |
| CT | 869 | -0.20 (-0.35, -0.05) | 0.011 |  |
| TT | 486 | -1.19 (-1.77, -0.62) | < 0.001 |  |

Adjusted for sex, age, study site, BMI, systolic blood pressure, diastolic blood pressure, estimated glomerular filtration rate, total cholesterol, triglycerides, high-density lipoprotein cholesterol, vitamin B12, vitamin D3, fasting glucose, folate, smoking and drinking at baseline.
